# Impact of Different Regimens of Fluoridated Dentifrice Application on the pH and Inorganic Composition in an Oral Microcosm Biofilm Model

**DOI:** 10.3390/microorganisms13071612

**Published:** 2025-07-09

**Authors:** Patrícia de Lourdes Budoia de Carvalho, Juliano Pelim Pessan, Bruna do Amaral, Amanda Costa Troncha, Samuel Campos Sousa, Douglas Roberto Monteiro, Thayse Yumi Hosida, Alberto Carlos Botazzo Delbem, Caio Sampaio

**Affiliations:** 1 Department of Pediatric Dentistry and Public Health, School of Dentistry, São Paulo State University (UNESP), Araçatuba 16015-050, SP, Brazil; paty.budooa@gmail.com (P.d.L.B.d.C.); juliano.pessan@unesp.br (J.P.P.); bruna.amaral@unesp.br (B.d.A.); amanda.troncha@unesp.br (A.C.T.); samuel.campos@unesp.br (S.C.S.); thayse.hosida@unesp.br (T.Y.H.); alberto.delbem@unesp.br (A.C.B.D.); 2 Department of Diagnosis and Surgery, School of Dentistry, São Paulo State University (UNESP), Araçatuba 16015-050, SP, Brazil; douglas.monteiro@unesp.br

**Keywords:** fluorides, biofilms, toothpastes, calcium, saliva, child, preventive dentistry

## Abstract

This study evaluated the pH, fluoride (F), and calcium (Ca) concentrations in saliva-derived microcosm biofilms following treatments with dentifrices applied at different amounts and F concentration. Human saliva was inoculated into McBain culture medium, and treatments were applied at 72/78/96 h (1 min). Fluoridated dentifrices containing 550 or 1100 ppm F (550F and 1100F, respectively) were used at the following combinations (intensities): (i-1) 550F/0.08 g or 1100F/0.04 g; (i-2) 550F/0.16 g or 1100F/0.08 g; (i-3) 550F/0.32 g or 1100F/0.16 g. A negative control (fluoride-free dentifrice—PLA) was also included. Biofilm F and Ca were measured with an ion-selective electrode and colorimetrically, respectively, while pH in the culture medium was measured with a pH electrode. Data were subjected to ANOVA and Student–Newman–Keuls’ test (*p* < 0.05). F-dentifrices did not significantly alter pH compared to PLA, except for 1100F at i-3. Biofilm F levels at i-1 and i-2 were comparable, for both 550F and 1100F, while 1100F at i-3 led to the highest biofilm F concentration. All F-groups showed significantly higher Ca levels than PLA, especially at i-2 and i-3. In conclusion, the interplay between dentifrice amount and F concentration was more influential on the biofilm’s inorganic composition and pH than either variable alone.

## 1. Introduction

Fluoridated dentifrices represent a widely used method of topical fluoride (F) delivery, with over 1.5 billion people using them worldwide [1]. Given the etiology of dental caries, toothbrushing with a fluoridated dentifrice is widely recognized as a fundamental approach for caries control, as it combines the F therapeutic effects on the demineralization–remineralization dynamics of dental tissues with the mechanical removal or disruption of dental biofilm [2,3,4]. This practice has played a significant role in the global decline of caries prevalence in recent decades [5,6].

Despite the substantial impact of fluoridated vehicles on caries control, excessive F intake, particularly from dentifrice, during the amelogenesis of permanent teeth (i.e., from ages 1 to 3, especially) has been closely related with the development of dental fluorosis [7]. In this context, various strategies have been proposed to minimize F ingestion from this source, including the use of low-fluoride dentifrices. Although such a recommendation is supported by the most recent Cochrane systematic review on the topic [8], this has frequently been subject of debate among researchers and scientific societies due to the reduced number trials assessing the effects of low-fluoride formulations (~550 ppm F) compared with those assessing conventional (~1000–1100 ppm F) ones. In this context, pediatric dentistry associations worldwide have recommended the use of fluoridated dentifrices at regular F concentrations (1000–1100 ppm F), but in reduced amounts [9,10,11]. However, a study evaluating oral hygiene guidelines for children issued by dental and pediatric organizations revealed considerable variation in the recommended amount of dentifrice [12]. Similarly, recent data have highlighted a considerable gap between caregivers’ understanding of these recommendations and the actual amount of dentifrice used [13,14,15]. Notably, factors such as parental oral health literacy seem to influence this pattern [16,17].

Based on the principle that the clinical effectiveness of fluoridated dentifrices is directly linked to the F available in the oral environment [18], researchers have investigated how factors such as amount of dentifrice and F concentration influence this availability. An in vivo study with children aged 8–10 years demonstrated that brushing with a 0.3 g of 550 ppm F dentifrice produced higher salivary F levels than brushing with 0.1 g of a 1100 ppm F formulation [19]. This underscores the importance of application technique and was further supported by a subsequent in vivo study in toddlers—a population especially vulnerable to dental fluorosis—reinforcing the clinical relevance of these findings [20].

In addition to the aforementioned patterns regarding intraoral F levels, several studies have reported that the concentration of F-treatments directly influences the pathogenicity and microbial profile of in vitro-formed cariogenic biofilms [21,22,23,24]. Recent findings indicate that the combined effect of dentifrice amount and F concentration has a greater impact than either factor alone on the inorganic composition of *Streptococcus mutans* and *Candida albicans* dual-biofilms formed under controlled conditions in 6-well polystyrene plates [25]. In that study, treatment with 550 ppm F applied at higher amounts led to significantly greater F levels in both the biofilm biomass and the fluid phase compared to 1100 ppm F applied in lower amounts [25].

Despite their relevance, such findings are limited by the fact that biofilms were grown under non-active conditions (i.e., statically, at the bottom of polystyrene plates), partially influenced by gravitational sedimentation. Moreover, dual-species biofilms fail to accurately replicate the structural and microbial complexity of oral biofilms under both healthy and cariogenic conditions [26]. Considering the critical role of dental biofilm in the caries process and the limited evidence regarding the interplay between dentifrice amount and F concentration on biofilm inorganic composition, a similar investigation using saliva-derived, multispecies biofilms could yield valuable insights into this topic. Such data could ultimately support the development of more robust, evidence-based recommendations for the use of fluoridated dentifrices in young children during the risk period for dental fluorosis, balancing both risks and benefits.

## 2. Materials and Methods

### 2.1. Ethics Aspects and Biofilm Formation

The study was approved by the Research Ethics Committee for Human Studies at the School of Dentistry of Araçatuba (CAAE 66163422.8.0000.5420), and all volunteers signed an informed consent form prior to participation. Human saliva was collected from five healthy volunteers using polypropylene tubes kept on ice during stimulated chewing with a flexible film (Parafilm^®^, Sigma-Aldrich, St. Louis, MO, USA). Participants were required to be in good general health and not taking medications that could interfere with biofilm formation or salivary flow [27]. Volunteers were instructed to refrain from toothbrushing both the night before and on the day of collection, and samples were obtained at least two hours after their last meal or drink. Additionally, participants refrained from consuming alcohol on the day prior to collection and were non-smokers.

Immediately after collection, individual saliva samples were pooled to form a single sample, which was diluted (1:1) with sterile 60% glycerol to protect microbial cells from cryodamage. Aliquots of 500 µL were transferred into sterile microtubes and stored at −80 °C until use [28]. To prepare the standardized microbial inoculum, the pooled saliva was thawed and diluted 1:50 in McBain culture medium supplemented with 0.2% sucrose [28,29]. The medium contained (per 1 L of deionized water): 2.5 g mucin (M2378, Sigma-Aldrich), 2.0 g Bacto peptone (Difco 0118-01-8), 2.0 g Trypticase peptone (BBL 211921), 1.0 g yeast extract (Bacto 212750), 0.35 g NaCl, 0.2 g KCl, 0.2 g CaCl₂, 0.001 g hemin (Sigma-Aldrich H1652), 0.0002 g vitamin K₁, 50 mmol/L PIPES buffer, and 0.2% sucrose, adjusted to pH 7.0 [28].

Biofilms were formed by adding the standardized inoculum to each well of a 24-well plate. The Amsterdam Active Attachment Model (AAA-model) was used to form biofilms adherent to glass coverslips, as described by Exterkate et al. [28]. Following inoculation, the biofilms were incubated in a 10% CO_2_ atmosphere at 37 °C for 96 h, with the culture medium refreshed daily. Treatments were performed at 72, 78, and 96 h (totaling three treatments), using experimental dentifrice slurries prepared by diluting the dentifrices in deionized water at a 1:3 ratio [25]. For each treatment, 2 mL of the slurry was pipetted into the wells of a 24-well microplate. The AAA-model lid was then positioned so that the glass coverslips were fully immersed in the slurry for 1 min. After each treatment, the disks were rinsed twice by immersion in wells containing 0.85% saline solution (NaCl) to remove residual dentifrice and then transferred to a new 24-well plate with fresh culture medium. Fluoridated dentifrices containing 550 or 1100 ppm F (550F and 1100F, respectively) were used as treatments in the following combinations: (i-1) 550F/0.08 g or 1100F/0.04 g; (i-2) 550F/0.16 g or 1100F/0.08 g; (i-3) 550F/0.32 g or 1100F/0.16 g. A fluoride-free dentifrice (0.32 g) served as the negative control (placebo; PLA). Further details on the experimental groups are presented in Table 1.

The dentifrices used in this study were the same formulations previously employed by Sampaio et al. [25]. The dentifrices were formulated in the Pediatric Dentistry Laboratory at São Paulo State University (UNESP), School of Dentistry, Araçatuba, Brazil, using the following ingredients: titanium dioxide, carboxymethyl cellulose, methyl p-hydroxybenzoate, sodium saccharin, peppermint oil, glycerine, silica abrasive, and sodium lauryl sulfate, water. In addition, sodium fluoride (NaF; Merck^®^, Darmstadt, Germany) at eighter concentrations of 550 or 1100 ppm F were used. A fluoride-free dentifrice (placebo) was also prepared. F concentrations (total and ionic) were measured using a F ion-selective electrode (Orion 9409 BN; Orion Research Inc., Beverly, MA, USA) connected to an ion analyzer (SevenCompact S220; Mettler Toledo, Greifensee, Switzerland), calibrated with standard solutions ranging from 0.125 to 2.0 ppm F, as follows. Approximately 100–110 mg of each dentifrice was placed into a test tube, followed by the addition of 10.0 mL of deionized water. The suspension was vigorously mixed to ensure homogeneity. For total F determination, a 0.25 mL aliquot of the suspension was taken and mixed with 0.25 mL of 2 mol/L hydrochloric acid (HCl). This mixture was kept under constant agitation at 45 °C for 1 h. After this period, 0.5 mL of 1 mol/L sodium hydroxide (NaOH) and 1.0 mL of TISAB II (total ionic strength adjustment buffer) were added. The remaining suspension was centrifuged at 906× *g* for 20 min at room temperature. For ionic F analysis, a 0.25 mL aliquot of the supernatant was combined with 0.25 mL of 2 mol/L HCl, 0.5 mL of 1 mol/L NaOH, and 1.0 mL of TISAB II [20,25]. The pH of the dentifrices was analyzed using a pH electrode (Accumet 13-620-290, Fisher Scientific, San Diego, CA, USA) calibrated with pH 4 and 7 standard solutions [20]. The dentifrice labeled as 550 ppm F presented mean (standard deviation) ionic and total F concentrations of 539.4 (44.1) ppm and 597.2 (21.1) ppm, respectively. For the 1100 ppm F formulation, the corresponding values were 1149.7 (24.1) ppm (ionic) and 1174.3 (35.4) ppm (total). The F-free dentifrice (placebo) showed residual ionic and total F levels of 11.9 (2.1) ppm and 12.7 (2.8) ppm, respectively [25]. The mean (standard deviations) pH of the dentifrices was 7.4 (0.2), 7.3 (0.3), and 7.4 (0.2), respectively, for the F-free, 550 ppm F, and 1100 ppm F dentifrice formulations.

### 2.2. Analytical Procedures

Biofilm pH was measured in the culture medium prior to the last treatment using a micro pH electrode (Accumet 13-620-290), previously calibrated with standards at pH 4.0 and 7.0 [30]. Following the final treatment, the biofilms were rinsed twice, as previously described [25]. The glass coverslips containing the biofilms were then carefully removed from the model using sterile tweezers and placed into 10 mL polystyrene tubes. For F and Ca analysis, 0.5 mol/L hydrochloric acid (HCl) was added to the microtubes containing the biofilm-coated coverslips, followed by homogenization [31]. The mixture was then maintained at ambient temperature (120 rpm; under constant agitation) for 3 h and subsequently transferred to microcentrifuge tubes and centrifuged at 11,000× *g* for 1 min [32]. A known volume of the supernatant was collected and immediately neutralized with an equal volume of 0.5 mol/L sodium hydroxide (NaOH).

F concentrations were analyzed using an ion-selective electrode (Orion 9409 BN) and a reference electrode (Orion 900100), both connected to a potentiometer (Orion—Thermo Scientific, Waltham, MA USA). Calibration curves were prepared using fluoride standards of 0.09, 0.18, 0.36, 0.72, and 1.44 ppm F for F-free treatment groups, and 0.8, 1.6, 3.2, 6.4, and 12.8 ppm F for F-containing treatments [30]. A total ionic strength adjustment buffer (TISAB II) was added to all samples and standards in a 1:1 ratio, under the same conditions.

Ca levels were analyzed via spectrophotometry using a microplate reader set at 650 nm [33]. Briefly, Arsenazo III colorimetric reagent was used. Duplicate aliquots of 5 μL from standards and samples were mixed with 50 μL of Arsenazo III and 50 μL of deionized water. The mixtures were agitated for 60 s in the microplate reader to allow the reaction to proceed before absorbance readings were recorded.

### 2.3. Statistical Analysis

The experiments were performed in triplicate across three independent runs (*n* = 9 samples per group). After assessing normality using the Shapiro–Wilk test, the data were subjected to ANOVA, followed by the Student–Newman–Keuls test, with a significance level of 5%. Statistical analysis was performed using SigmaPlot 12.0 software.

## 3. Results

**Figure 1:**
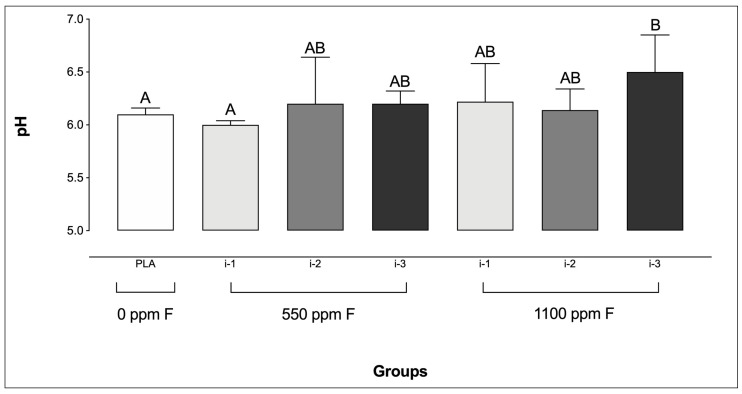
pH of the culture medium prior to the final treatment in saliva-derived microcosm biofilms across the experimental groups. Different letters denote statistically significant differences among the groups (ANOVA, Student–Newman–Keuls’ test, *p* < 0.05; *n* = 9). PLA = fluoride-free dentifrice applied at 0.32 g.

Only biofilms treated with 1100 ppm F applied at the highest amount (i-3) exhibited significantly higher pH values compared to the PLA group. Additionally, no significant differences were found among the F-treated groups, except between 550 ppm F at i-1 and 1100 ppm F at i-3 (Figure 1).

**Figure 2:**
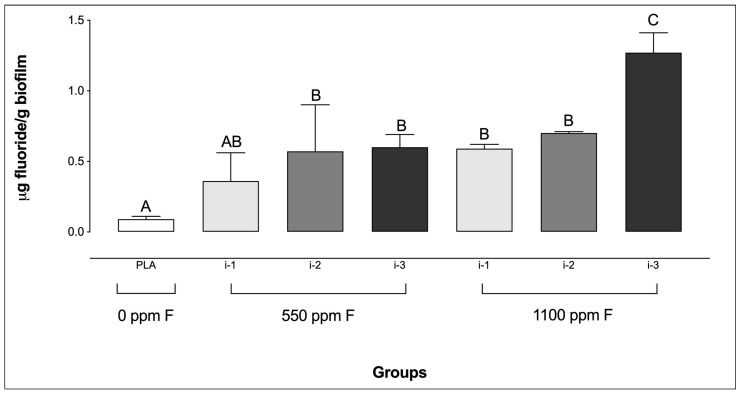
Fluoride concentrations in saliva-derived microcosm biofilms after treatments with dentifrices of different fluoride concentrations, administered in varying quantities. Different letters denote statistically significant differences among the groups (ANOVA, Student–Newman–Keuls’ test, *p* < 0.05; *n* = 9). PLA = fluoride-free dentifrice applied at 0.32 g.

Regarding biofilm F concentrations, the placebo group showed the lowest levels, with no significant difference compared to the 550 ppm F group at intensity i-1. In contrast, the highest F concentrations were observed in the 1100 ppm F group at intensity i-3 (Figure 2). It is noteworthy that no significant differences were observed among the F-containing dentifrice groups at i-1 and i-2, regardless of the dentifrice F concentration or the amount used.

**Figure 3:**
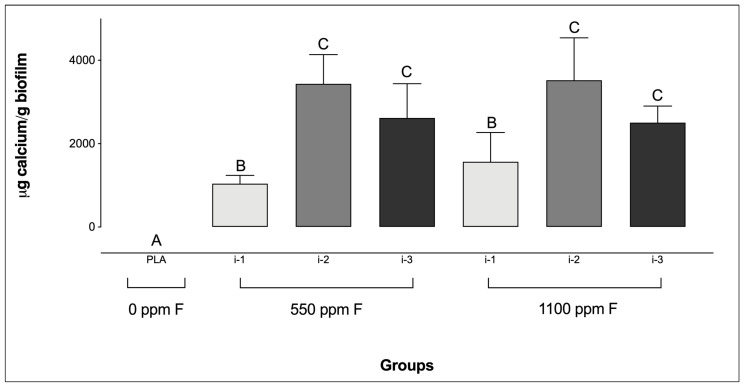
Calcium concentrations in saliva-derived microcosm biofilms after treatments with dentifrices of different fluoride concentrations, administered in varying quantities. Different letters denote statistically significant differences among the groups (ANOVA, Student–Newman–Keuls’ test, *p* < 0.05; *n* = 9). PLA = fluoride-free dentifrice applied at 0.32 g.

Regarding Ca levels, all F-containing dentifrices, regardless of the amount applied, resulted in significantly higher Ca concentrations compared to the PLA group (Figure 3). Additionally, for the F-dentifrices, biofilms treated at intensity i-1 exhibited significantly lower Ca concentrations than those treated at i-2 or i-3, regardless of the F concentration of the dentifrices. No significant differences were observed between groups at intensities i-2 and i-3, irrespective of either the F concentration or the amount of dentifrice used.

## 4. Discussion

Although recent findings have provided valuable insights into the interplay between dentifrice amount and F concentration on the inorganic composition and pH of cariogenic-related biofilms [25], the experimental model employed in that study presented certain limitations mainly related to the use of a dual-species biofilm (*C. albicans* and *S. mutans*) formed passively. To overcome these limitations, the present study aimed to evaluate similar parameters in saliva-derived microcosm biofilms (i.e., polymicrobial in nature), formed under active adhesion conditions using the AAA-model [28]. The results demonstrated that the interplay between dentifrice amount and F concentration was more influential on the biofilm’s inorganic composition and pH than either variable alone, leading to the rejection of the study’s null hypothesis.

Consistent with previous findings by Sampaio et al. [25], this study showed that the use of a dentifrice containing 1100 ppm F applied at the highest amount (i-3) resulted in the greatest F concentrations in biofilms when compared to all other groups, including its 550 ppm F counterpart at the same intensity (i-3). Although similar F concentrations might have been expected for both 550 and 1100 ppm F groups at intensity i-3 (due to the similar F concentrations in the respective slurries), the observed differences could be explained by the distribution profile of F within biofilm layers. Using a methodology that allows F determination in serial sections of biofilms (i.e., Leeds in situ device), previous studies demonstrated that the treatment of polymicrobial biofilms formed in situ with a fluoridated solution led to F accumulation in the outer sections [34,35,36]. Additionally, F distribution across biofilm layers after exposure to fluoridated dentifrices has also been investigated using the same methodology [37], further supporting that F remains mostly confined to superficial layers. Based on these findings, it can be hypothesized that the viscosity of the dentifrice may limit F diffusion through the biofilm, since biomass density increases from the biofilm–saliva interface towards its deeper regions, while the availability of channels and voids decreases in the same direction [38]. Moreover, evidence from non-dental biofilm models suggests that solute transport through channels is considerably faster than through dense biomass due to reduced diffusional resistance [39]. In this sense, the higher density of 550F at i3 (using 0.32 g of dentifrice) compared with that of 1100 ppm F at i3 (using 0.16 g of dentifrice) might have somehow limited F penetration into the biofilms in the present study. While these hypotheses are plausible, further investigation is warranted under microbiological conditions similar to those used in the present study.

Another noteworthy finding concerning F concentrations in the biofilms was the lack of significant differences between the group treated with 550 ppm F at the lowest intensity (i-1) and the placebo group. Although these results differ from those reported by Sampaio et al. [25], who observed a significant difference between these two groups, they are consistent with findings from Sampaio et al. [20] in an in vivo study involving toddlers. In that instance, brushing with a 550 ppm F dentifrice applied at 0.04 g led to salivary F concentrations comparable to those obtained with a F-free (placebo) dentifrice. These findings are highly relevant, as they reinforce the concept that the use of very small amounts of dentifrice may substantially limit F uptake in oral compartments and/or the biofilm, potentially reducing the preventive effectiveness of the product.

Beyond the points discussed above, an important observation is that no significant differences were found between the two fluoridated dentifrice concentrations at intensities 1 and 2 regarding biofilm F levels. This aligns with prior in vivo findings showing that below 0.16 g, 1100 ppm F did not result in significantly higher intraoral F levels than 550 ppm F [20], suggesting that at very small quantities, product performance is comparable regardless of F concentration, thus supporting the concept that minimal dentifrice amounts may limit F uptake. These findings have potential clinical implications. Current pediatric dentistry guidelines [9,10,11] recommend using no more than a “rice-grain” amount of a ~1000–1100 ppm F dentifrice for children under 3 years old, progressively increasing up to a “pea-sized” amount for those under 6 years old.

Interestingly, the F concentration patterns described above were generally reflected in the biofilm pH values following treatment. Notably, 1100 ppm F at intensity 3 was the only condition that resulted in significantly higher pH values compared to the placebo. The mechanism by which F affects microbial acid production has been previously described, showing that F acts on the microbial glycolytic pathway, thereby reducing acid production by the biofilm [40]. In brief, under low pH conditions, hydrogen fluoride (HF) diffuses across the bacterial cell membrane and dissociates into H^+^ and F^−^ in the cytoplasm, which is more alkaline than the external environment [40]. The intracellular F^−^ then inhibits glycolytic enzyme activity, leading to reduced acid generation from glycolysis. Furthermore, the presence of F^−^ in the cytoplasm also contributes to a decrease in cytoplasmic pH by impairing glycolytic activity, thereby affecting both acid production and acid tolerance mechanisms [41,42].

Although it was expected that all treatments with fluoridated dentifrices would maintain higher biofilm pH values compared to the placebo, several factors may have influenced the patterns observed in the present study. First, unlike the protocol used by Sampaio et al. [25], in which biofilms were formed and treated at the bottom of polystyrene plates, the present biofilms were formed under active adhesion conditions on glass coverslips. As such, the penetration of active compounds into the biofilm layers was not influenced by gravity, relying instead on the physicochemical properties of the biofilms and the treatment suspensions. Another important distinction between protocols lies in the handling of the active attachment model. In the present study, the model was transferred between plates for treatment and incubation steps, whereas in the study by Sampaio et al. [25], the biofilms remained in the same plate throughout the entire experimental period. This difference may have increased the likelihood of residual treatment slurry or rinse solution remaining in the wells in the earlier study, potentially influencing the pH values observed. Therefore, the differing pH trends reinforce the importance of forming biofilms under active adhesion in studies of this nature, as it may provide more accurate and clinically relevant results.

With respect to Ca, biofilms treated with fluoridated dentifrices exhibited significantly higher Ca concentrations compared to the placebo group. Moreover, Ca levels were significantly greater in intensities i-2 and i-3 compared to i-1, regardless of the F concentration in the dentifrice, in agreement with previous findings [43,44,45,46,47]. In summary, F is attracted as a counter-ion to Ca bound to acidic groups adhered to the biofilm matrix [46,47]. Interestingly, the data from the present study contrast with those reported by Sampaio et al. [25], in which treatment with a F-free dentifrice tended to result in higher Ca values than its fluoridated counterpart, particularly in the biofilm fluid. Similarly to the trends observed for pH, differences in biofilm formation protocols may have influenced these outcomes. Thus, although the present study did not incorporate dynamic oral variables such as salivary clearance, frequently considered in in vivo protocols, the fact that all experimental procedures were conducted under active conditions suggests that the influence of intrinsic oral dynamics may have been attenuated compared to non-active biofilm formation methods.

Despite the valuable insights provided by this study regarding the inorganic composition and pH of biofilms following different fluoridated dentifrice regimens, some limitations must be acknowledged. Notably, the protocol did not simulate salivary clearance, a key variable in the dynamics of the oral environment [48]. This limitation restricts the extrapolation of the results to in vivo conditions. Additionally, the absence of a mineral substrate limits deeper interpretations, as the inorganic composition analyzed reflects only the interactions among the treatments, the culture medium, and the biofilm itself. In this context, future studies that incorporate these variables could yield more comprehensive data on ionic exchanges among the biofilm, the oral environment, and the mineral substrate, while evaluating different dentifrice application protocols that vary in both F concentration and amount used. Finally, it is important to highlight that aspects such as different rinsing protocols, types of F salts as well as the inclusion of other active ingredients may exert important role in the dynamics investigated in the present study [49,50,51,52,53,54]. Therefore, exploring these factors in future research could provide valuable additional insights and further strengthen our current understanding of the topic. It should be emphasized, however, that the aforementioned limitations were also instrumental in enabling strict control over experimental variables, thus facilitating a better understanding of the underlying mechanisms.

## 5. Conclusions

Based on the findings, it can be concluded that the interplay between dentifrice amount and F concentration in the product was more influential on the biofilm’s inorganic composition and pH than either variable alone. Although the present results are consistent with the previous literature, it is important to emphasize that the in vitro nature of the protocol employed should be taken into account when interpreting the results and extrapolating them to clinical conditions.

## Figures and Tables

**Table 1:** Study groups according to the amount of dentifrice used and fluoride concentration of the dentifrices.

Fluoride Concentration (ppm F)	Amount of Dentifrice (g)	Intensity
Fluoride-free (placebo)	0.32	Control
550	0.08	Intensity 1 (i-1)
1100	0.04	
550	0.16	Intensity 2 (i-2)
1100	0.08	
550	0.32	Intensity 3 (i-3)
1100	0.16	

## Data Availability

The original contributions presented in this study are included in the article. Further inquiries can be directed to the corresponding author.
